# Analytical and experimental FWHM of a gamma camera: theoretical and practical issues

**DOI:** 10.7717/peerj.722

**Published:** 2015-02-03

**Authors:** Diego Cecchin, Davide Poggiali, Lucia Riccardi, Paolo Turco, Franco Bui, Stefano De Marchi

**Affiliations:** 1 Department of Medicine DIMED, University-Hospital of Padova, Italy; 2 Department of Mathematics, University of Padova, Italy; 3 Medical Physics Unit, Veneto Institute of Oncology IOV-IRCCS, Italy

**Keywords:** Gaussian, Splines, Gamma camera, FWHM, Resolution, Local interpolation, Global interpolation

## Abstract

**Introduction.** It is well known that resolution on a gamma camera varies as a function of distance, scatter and the camera’s characteristics (collimator type, crystal thickness, intrinsic resolution etc). Manufacturers frequently provide only a few pre-calculated resolution values (using a line source in air, 10–15 cm from the collimator surface and without scattering). However, these are typically not obtained in situations resembling a clinical setting. From a diagnostic point of view, it is useful to know the expected resolution of a gamma camera at a given distance from the collimator surface for a particular setting in order to decide whether it is worth scanning patients with “small lesion” or not. When dealing with absolute quantification it is also mandatory to know precisely the expected resolution and its uncertainty in order to make appropriate corrections.

**Aim.** Our aims are: to test a novel mathematical approach, the cubic spline interpolation, for the extraction of the full width at half maximum (FWHM) from the acquisition of a line source (experimental resolution) also considering measurement uncertainty; to compare it with the usually adopted methods such as the gaussian approach; to compare it with the theoretical resolution (analytical resolution) of a gamma camera at different distances; to create a web-based educational program with which to test these theories.

**Methods.** Three mathematical methods (direct calculation, global interpolation using gaussian and local interpolation using splines) for calculating FWHM from a line source (planar scintigraphy) were tested and compared. A NEMA Triple Line Source Phantom was used to obtain static images both in air and with different scattering levels. An advanced, open-source software (MATLAB/Octave and PHP based) was created “ad hoc” to obtain and compare FWHM values and relative uncertainty.

**Results and Conclusion.** Local interpolation using splines proved faster and more reliable than the usually-adopted Gaussian interpolation. The proposed freely available software proved effective in assessing both FWHM and its uncertainty.

## Introduction

The spatial resolution of a gamma camera is a measure of its ability to resolve small objects in the field of view. Spatial resolution can also be defined as the minimum distance between two points such that they can be pictured separately. This means that objects placed at a distance smaller than the resolution limit are imaged as a single blurred one.

Minute variations (even of 0.3 mm) in the system’s resolution could affect image quality (Dendy, Barber & Bayliss, 1988). Therefore, it is important to know precisely “a priori” what the gamma camera’s limits are before scanning a patient. Moreover, when dealing with the absolute quantification of the tracer in SPECT, the measure of the mean radioactivity in a volume of interest (VOI) can be affected by an error proportional to the resolution (Kojima et al., 1989). Resolution is therefore a crucial parameter that measures the reliability of the gamma camera in a specific setting (Soreson & Phelps, 1987). A precise measure of resolution, together with the uncertainty of such measure, can lead to an appropriate qualitative reading of images and to a correct quantitative evaluation.

The overall spatial resolution of a gamma camera system (*R_s_*) depends on different factors, both geometrical and physical, and it is usually expressed in terms of collimator resolution (*R_c_*) and intrinsic resolution (*R_i_*). The *R_s_* is typically assessed from the full width at half maximum (FWHM) of the profile of a point-like, or line-like, radiation source.

FWHM can be expressed as a function of the gamma camera’s characteristics and the distance between the object and the collimator (Soreson & Phelps, 1987; Cherry, Sorenson & Phelps, 2012; Zaidi, 2006) (the so-called *analytical resolution*) or computed from the experimental data obtained from the image of a line source (the so-called *experimental resolution*).

Different methods have been proposed to calculate the experimental FWHM from a point spread function (PSF) or line spread function (LSF) (Hander et al., 1997; Hander et al., 2000; Wasserman, 1998; Metz, Atkins & Beck, 1980), but none of these methods provide the uncertainty of the measure of FWHM.

The aim of this work is to introduce a method for computing FWHM (using a matematical method known as splines) in the case of a parallel-hole collimator and the relative uncertainty from a LSF and compare it to the usually adopted methods. The most reliable one will be chosen using a cost function.

Every algorithm described in this paper was implemented and tested on the Phantom’s data and is part of the freely-available package (Resolution_Calculator_0.1.zip) developped by our group for educational purposes: http://www.rad.unipd.it/fwhm/.

### Analytical resolution

The system resolution *R_s_* depends on the *collimator resolution* *R_c_* and on the *intrinsic resolution* *R_i_* (Soreson & Phelps, 1987; Cherry, Sorenson & Phelps, 2012; Zaidi, 2006). Using the convolution mathematical theory (Cherry, Sorenson & Phelps, 2012), we obtain ${R}_{s}^{2}={R}_{c}^{2}+{R}_{i}^{2}$, which gives us

(1) \begin{eqnarray*} \displaystyle {R}_{s}=\sqrt{{R}_{c}^{2}+{R}_{i}^{2}}\hspace{0.167em} &&\displaystyle \end{eqnarray*}

due to the fact that *R_s_* will be positive.

The intrinsic resolution *R_i_* is linked to the properties of the detector and electronics. For the given energy of a photon, *R_i_* could be considered independent of the object-to-collimator distance, whereas the collimator‘s resolution depends largely on the geometrical layout and can be expressed as a function of a number of parameters:

• *x*: distance between the object and the collimator’s surface;
• *L*: collimator’s hole length;
• *D*: collimator’s hole size;
• *c*: crystal’s thickness, including an estimate of the gap between collimator and crystal and between crystal and image plane. An estimate of the average depth of interaction in the crystal has also been considered;
• *t*: thickness of the septa

where *L*, *D*, *c*, *t* are declared by the manufacturer as well as *R_i_*.

**Figure 1:**
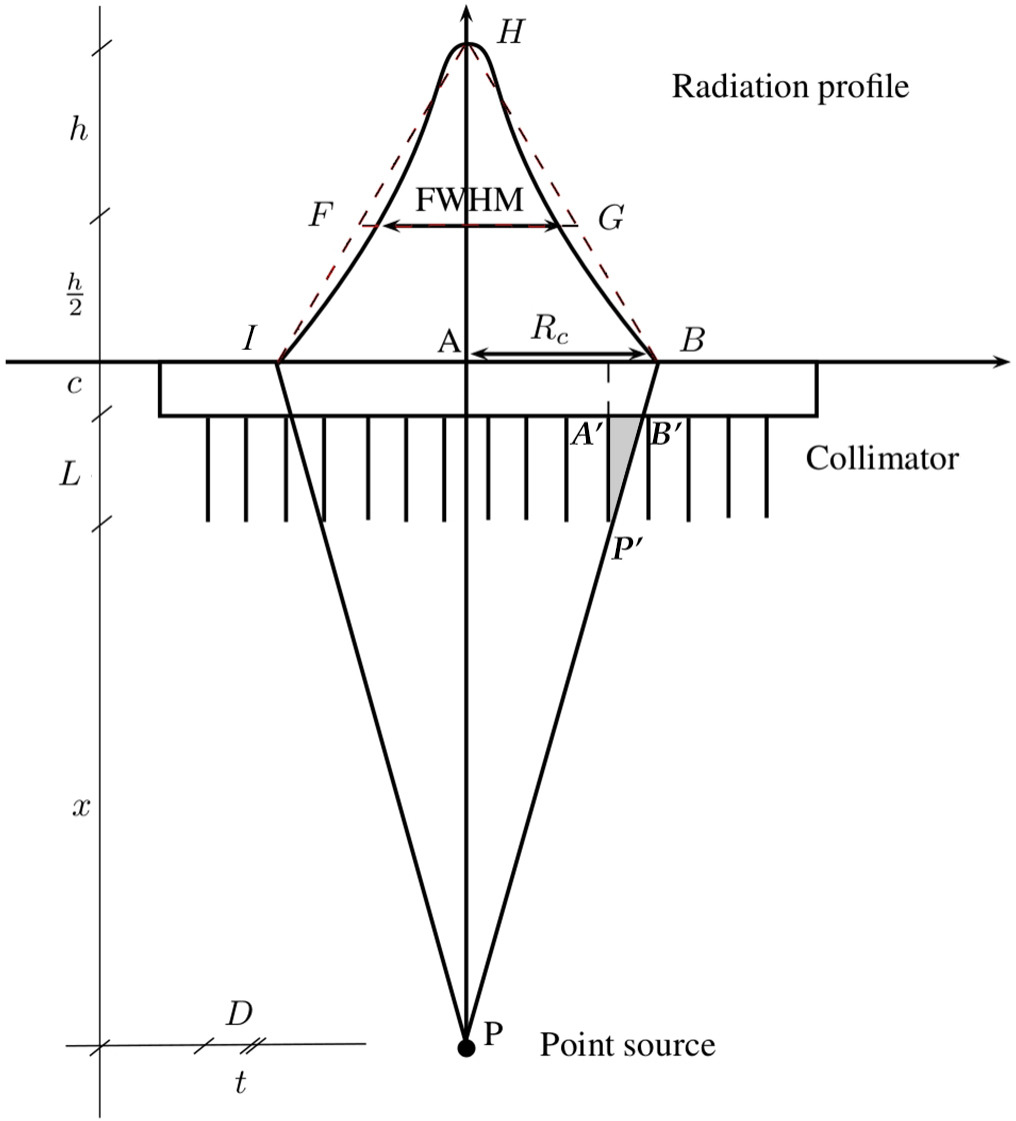
Geometrical layout of a point source acquisition by a gamma camera equipped with a parallel hole collimator (*D* is the diameter of the holes of the collimator and t is the septal thickness).

Figure 1 schematically shows the geometrical layout of a point source.

To calculate *R_c_*, consider gamma rays coming from a point source *P* (as in Fig. 1) and particularly rays $\overline{P A}$ (parallel to the septa) and $\overline{P B}$ (angular limit). Now, since the radiation profile is similar in shape to an isosceles triangle ($\mathop { H I B}\limits ^{\bigtriangleup }$) and triangles $\mathop { H I B}\limits ^{\bigtriangleup }$ and $\mathop { H F G}\limits ^{\bigtriangleup }$ are similar, then the FWHM is about half of the base $({R}_{c}\simeq \overline{A B}$).

Because of the similitude of the triangles $\mathop { P A B}\limits ^{\bigtriangleup }$ and $\mathop { {P}^{{\prime}}{A}^{{\prime}}B}\limits ^{\bigtriangleup }$ and the fact that $t\ll D(=\overline{{A}^{{\prime}}{B}^{{\prime}}})$ we infer that

\begin{eqnarray*} \overline{{P}^{{\prime}}{A}^{{\prime}}}:\overline{{A}^{{\prime}}{B}^{{\prime}}}=\overline{P A}:\overline{A B} \end{eqnarray*}

which is equivalent to:

(2) \begin{eqnarray*} \displaystyle L:D=(c+L+x):{R}_{c}.&&\displaystyle \end{eqnarray*}

Instead of *L*, *L_eff_* is usually used which is a length that is weighted to take the septal penetration into account,

(3) \begin{eqnarray*} \displaystyle {L}_{e f f}=L-\frac{2}{\mu }.&&\displaystyle \end{eqnarray*}

The constant *μ* is the linear attenuation coefficient of the material of the collimators (usually lead, *μ* = 2.49 mm^−1^ at 140 KeV). Thus from (2) and (3):

(4) \begin{eqnarray*} \displaystyle {R}_{c}=D\left(1+\frac{x+c}{{L}_{e f f}}\right).&&\displaystyle \end{eqnarray*}

## Materials and Methods

### Image acquisition

**Figure 2:**
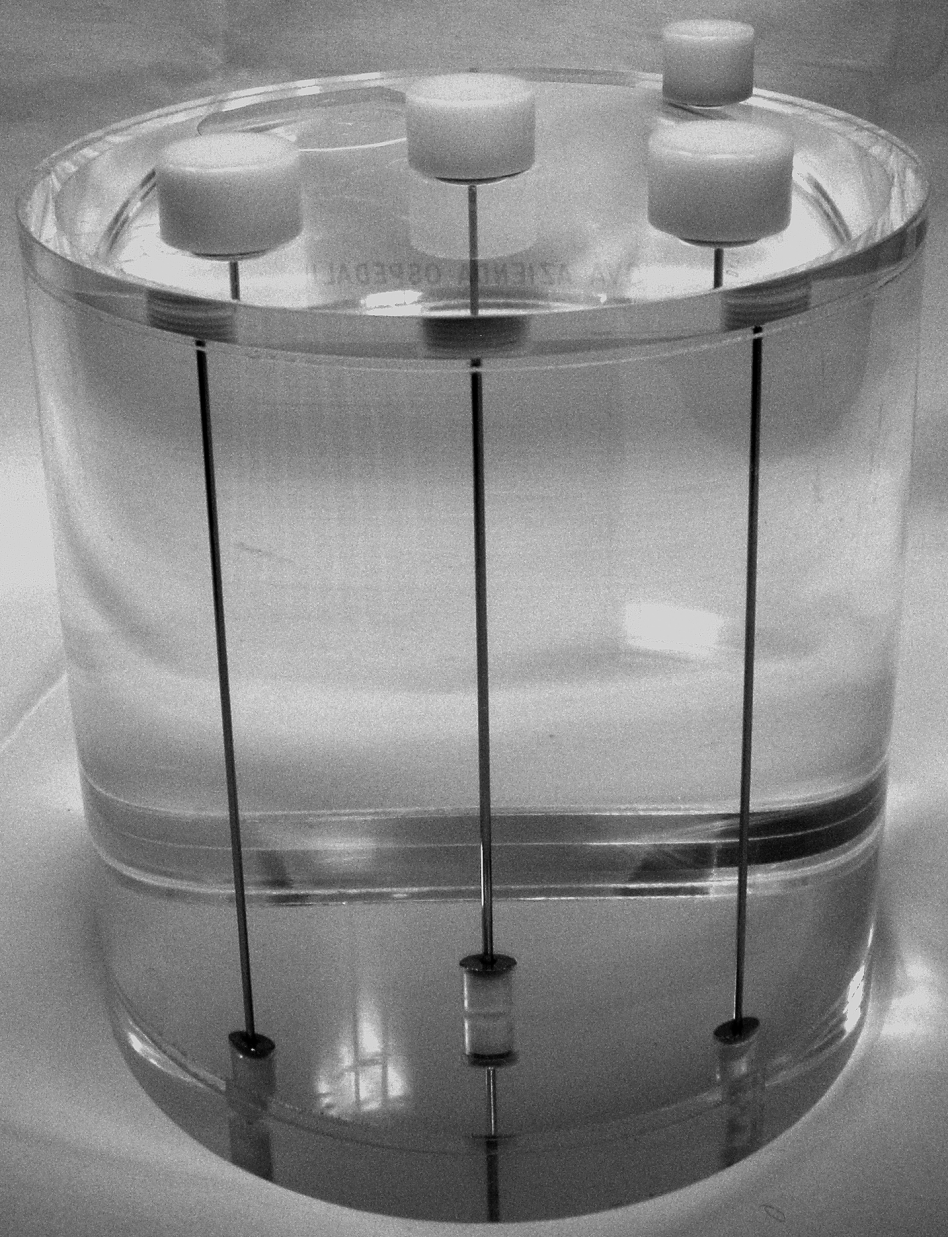
NEMA SPECT Triple Line Source Phantom used in the experiments (photograph by Diego Cecchin).

A planar static image of a line source, filled with about 200 MBq of ^99*m*^*Tc* activity and inserted in the center of a *NEMA SPECT Triple Line Source Phantom* (as in Fig. 2) was acquired using a Thriple-Head Irix Marconi-Philips gamma-camera (256 × 256 matrix, 180 s) equipped with a parallel-hole, ultra-high resolution collimator. The line source was placed in air, water and a radioactive solution (about 30 kBq/ml of ^99*m*^*Tc*) to reproduce different background conditions. A loss of resolution (Cherry, Sorenson & Phelps, 2012) was expected as a consequence of an increasing scattering effect. Planar images were acquired with the Phantom at increasing source-to-collimator distances (134, 164, 194, 224, 254 and 284 mm) and exported in DICOM format.

### From data to experimental resolution

**Figure 3:**
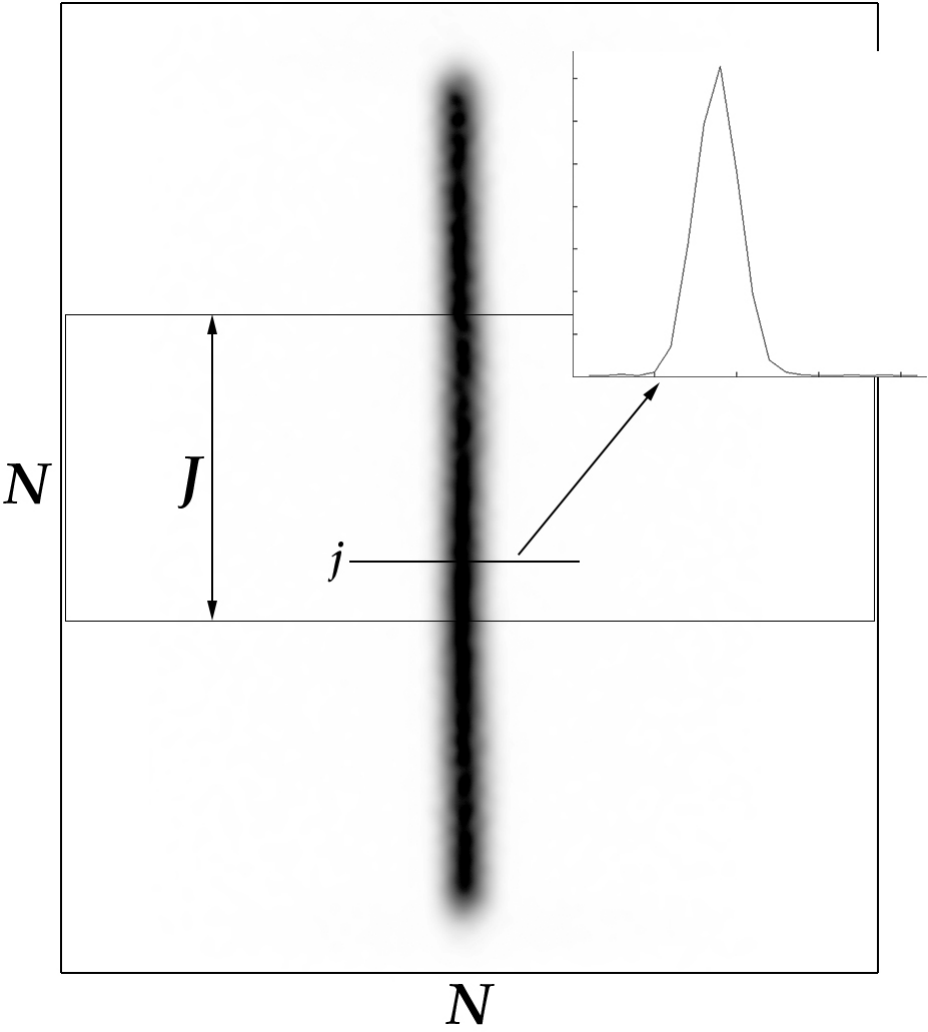
Line source acquired using “NEMA SPECT Triple Line Source Phantom” (in inverted gray scale) and a plot of activity at a certain height *j*∈*J*.

Figure 3 shows an image derived from the acquisition of a line source. It is an *N* × *N* data matrix with the number of radioactive counts in *N* points at *N* different heights. For each image, an *N* × *J* submatrix was visually selected (on the middle third of the line) so as to obtain near-constant data profiles For each j-th row of the submatrix, *FWHM_j_* was calculated from the data (*x_i_*,*y_i_*)_*i*=1,…,*N*_ using the three methods described below.

The FWHM value was assessed as the average of *FWHM_j_* (*j* = 1, …, *J*).

The standard deviation *σ* and the *variation coefficient* *Cv* were calculated to estimate the absolute and relative uncertainties respectively where

\begin{eqnarray*} C v=\frac{100\cdot \sigma }{F W H M}. \end{eqnarray*}

Another way to quantify the uncertainty of FWHM is the use of a quadratic *cost* defined case by case. The maximum error in FWHM is expected to be proportional to the square root of such cost (Walter & Pronzato, 1997).

#### Method 1: direct calculation

This first method was intentionally a basic one to prove that a naive approach will lead to an unreliable FWHM value.

The maximum pixel value *h* = max(*y_i_*) = *y_K_* for a proper index *K* and its argument $\tilde {x}={x}_{K}$ were found. Two points ${z}_{1}\lt \tilde {x}$ and ${z}_{2}\gt \tilde {x}$, which are the closest to $\frac{h}{2}$, were used and their distance

\begin{eqnarray*} F W H{M}_{j}=\vert {z}_{1}-{z}_{2}\vert \end{eqnarray*}

was ascertained.

For this case the following cost was defined:

(5) \begin{eqnarray*} \displaystyle {C}_{1}({z}_{1},{z}_{2})=\frac{(y({z}_{1})-h/2)^{2}+(y({z}_{2})-h/2)^{2}}{2}.&&\displaystyle \end{eqnarray*}

#### Method 2: Gaussian—global interpolation

Data (*x_i_*, *y_i_*) were modelled as a deterministic function with a small level of noise. The process called *least-squares* is reliable for choosing a function close to the data. Mathematically speaking, the least-squares approximation of a given data set looks for the best fit minimizing a suitable functional. Usually the functional is the sum of the squares of all deviations of a function chosen from the data.

The linear least-squares approximation consists in finding a function ${f}_{\bar {a}}(x)=\sum _{i=1}^{n}{a}_{i}{\phi }_{i}(x)$ depending on some parameters $\bar {a}=({a}_{1},\ldots ,{a}_{n})$, with *ϕ_i_*, *i* = 1, …, *n* a set of known (basis) functions. Nonlinear least-squares approximation can also be constructed (see below), providing a function ${f}_{\bar {a}}$ that is a nonlinear combination of some known functions.

The algorithm looks for:

\begin{eqnarray*} {\bar {a}}^{\ast }=\arg \nolimits \min _{\bar {a}\in \mathbb{R}^{n}}J(\bar {a})=\arg \nolimits \min _{\bar {a}\in \mathbb{R}^{n}}\Vert {y}_{i}-{f}_{\bar {a}}({x}_{i})\Vert ^{2}. \end{eqnarray*}

Since ${f}_{\bar {a}}$ is not always linearly dependent on the parameters $\bar {a}$, an iterative optimization algorithm was used to estimate ${\bar {a}}^{\ast }$. If the Optimization Toolbox/optim package is installed in MATLAB/Octave, the proposed software will use the well-known Levenberg–Marquardt algorithm; if not, it will use the Gauss-Newton algorithm (Nocedal & Wright, 2006).

The cost used in this method, which is a mean square deviation (as the cost used in direct calculation) was

(6) \begin{eqnarray*} \displaystyle {C}_{2}(\bar {a})=\frac{J({\bar {a}}^{\ast })}{N}=\frac{\Vert y-{f}_{{\bar {a}}^{\ast }}(x)\Vert ^{2}}{N}.&&\displaystyle \end{eqnarray*}

The *Gaussian* function was used (Zaidi, 2006):

\begin{eqnarray*} {f}_{\bar {a}}(x)={a}_{1}{e}^{-{a}_{2}^{2}{x}^{2}} \end{eqnarray*}

which has a resolution $F W H{M}_{j}=2\frac{\sqrt{\log (2)}}{\vert {a}_{2}\vert }$.

#### Method 3: splines—local interpolation

The third method proposes the use of *splines* of degree 1 (linear), 2 (quadratic) or 3 (cubic) calculated on a huge number of points (we used 10^4^ interpolation points in the experiments).

A spline of order *m* is a function *s*(*x*) defined by the following (de Boor, 2001; Lancaster & Šalkauskas, 1986):

1. on each subinterval *I_i_* = [*x_i_*, *x*_*i*+1_], *i* = 1, …, *N*−1, *s*_|*I_i_*_ = *s_i_*∈𝒫^*m*^(ℝ) where 𝒫^*m*^(ℝ) is the space of real polynomials of degree ≤ *m*.
2. *s*(*x_i_*) = *y_i_*, *i* = 1, …, *N*, i.e., *s* interpolates the data.
3. ${s}_{i}^{(k)}({x}_{i})={s}_{i+1}^{(k)}({x}_{i}),i=2,\ldots ,N-1,k\leq m-1,m\leq N$. This means that the polynomial pieces are continuous up to order *m*−1 in each inner point.

This approach is called *local interpolation*. **Cubic splines** were chosen for their well-known approximation properties (de Boor, 2001), and for the ability to provide a model-independent interpolation. In other words, the cubic spline approach is able to accurately follow the shape suggested by discrete data on a pointwise basis instead of searching a global fitting function.

As in Method 1 the algorithm searches for two points *z*_1_ and *z*_2_ a minimal distance away from the half of the maximum:

\begin{eqnarray*} {z}_{i}=\arg \nolimits \min _{x\in J_{i}}\vert s(x)-h/2\vert ,\hspace{1em}i=1,2 \end{eqnarray*}

where *J*_1_ and *J*_2_ are sets of 10^4^ equidistant points of the intervals $({x}_{1},\tilde {x})$ and $(\tilde {x},{x}_{N})$ respectively.

The distance between these two points gives a good estimate of the *FWHM_j_*

\begin{eqnarray*} F W H{M}_{j}=\vert {z}_{1}-{z}_{2}\vert . \end{eqnarray*}

The cost is defined as in Method 1:

(7) \begin{eqnarray*} \displaystyle {C}_{3}({z}_{1},{z}_{2})=\frac{(s({z}_{1})-h/2)^{2}+(s({z}_{2})-h/2)^{2}}{2}.&&\displaystyle \end{eqnarray*}

### Computation of analytical and experimental resolution curves

On the basis of formulas (1) and (4), *R_s_* can be expressed as follows:

(8) \begin{eqnarray*} \displaystyle {R}_{s}(x)=\sqrt{{p}_{1}{x}^{2}+{p}_{2}x+{p}_{3}}.&&\displaystyle \end{eqnarray*}

Using parameters (*L*, *D*, *c*, *t*, *R_i_*) declared by the manufacturer (Table 1) the following values were calculated for the analytical resolution:

(9) \begin{eqnarray*} \displaystyle {p}_{1}\cong 0.0010,\qquad {p}_{2}\cong 0.1468,\qquad {p}_{3}\cong 2.4267&&\displaystyle \end{eqnarray*}

**Table 1:** Parameters declared by the manufacturer of the gamma camera used (Triple-Head Irix Marconi-Philips).

*L*	*D*	*c*	*t*	FWHM	FWHM	*R_i_*
(mm)	(mm)	(mm)	(mm)	@ 0 mm	@ 100 mm	(mm)
58.4	1.78	19	0.152	4.8 mm	6.7 mm	4.1

The formula (8) was also used to fit the experimental FWHM data obtaining an *experimental fitting curve*.

The parameters that describe the experimental fitting curve were computed using the *weighted least-squares method*, as defined in Lancaster & Šalkauskas (1986).

To caclulate parameters an expression equivalent to (8) was used:

\begin{eqnarray*} {y}^{2}={p}_{1}{x}^{2}+{p}_{2}x+{p}_{3} \end{eqnarray*}

where *y* is the vector of *FWHM* s and *y*^2^ is the pointwise square of *y*. The vector *p* = (*p*_1_, *p*_2_, *p*_3_) can be obtained by solving the normal equations of the weighted least squares method

\begin{eqnarray*} ({V}^{T}\Sigma V)p={V}^{T}\Sigma f \end{eqnarray*}

where *V* is the Vandermonde matrix, *f* = *y*^2^ and $\Sigma =d i a g(1/{\sigma }_{1}^{2},\ldots ,1/{\sigma }_{n}^{2})$ the weight matrix, and *σ_i_* is the standard deviation of the *i* th FWHM value.

Usually the manufacturer provides the FWHM values at 0 and 100 mm. These two values (not measurable in our experimental setting), which weigh 90% less than the experimental FWHM ones, were used to regularize the fitting curve only in a scatterless condition (in air).

## Results

The experimental results, obtained by scanning the phantom in air, are given in Table 2 where the absolute (*σ*) and relative (Cv) deviations between the experimental and analytical FWHM are also shown.^1^1The interested reader can use the data contained in our MATLAB/Octave package to replicate these results.

**Table 2:** Experimental results in air and analytical results.

**Experimental FWHM**							
	radius (mm)	134	164	194	224	254	284
Direct Met.	FWHM (mm)	7.31	8.13	8.96	9.71	10.62	11.37
	*σ* (mm)	0.80	1.19	0.87	0.87	1.18	0.80
	Cv (%)	10.9	14.6	9.7	9.0	11.1	7.0
	mean cost	147543	137062	88423	66376	44393	34223
Global Int.	FWHM (mm)	7.53	8.20	8.88	9.65	10.54	11.35
	*σ* (mm)	0.24	0.22	0.22	0.21	0.33	0.30
	Cv (%)	3.2	2.7	2.5	2.2	3.1	2.7
	mean cost	3.75	3.11	2.33	2.06	2.15	1.99
Local Int.	FWHM (mm)	7.50	8.23	8.92	9.70	10.54	11.36
	*σ* (mm)	0.26	0.27	0.26	0.26	0.37	0.31
	Cv (%)	3.4	3.3	2.9	2.6	3.5	2.7
	mean cost	1.55	0.95	0.79	0.49	0.35	0.25
**Analytical FWHM**							
	FWHM (mm)	7.70	8.50	9.33	10.17	11.03	11.89

**Figure 4:**
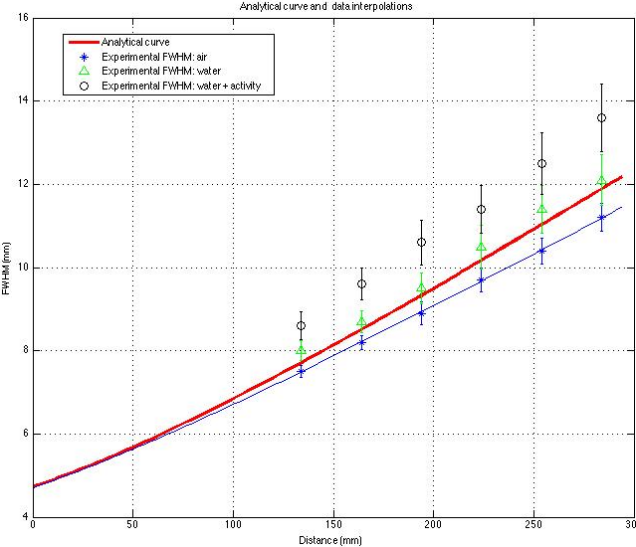
Experimental FWHM values, obtained using splines, in different scattering conditions. The analytical and fitting curves (in air) are also shown.

The analytical and experimental (both in air and with different level of background) FWHM curves are shown in Fig. 4.

**Figure 5:**
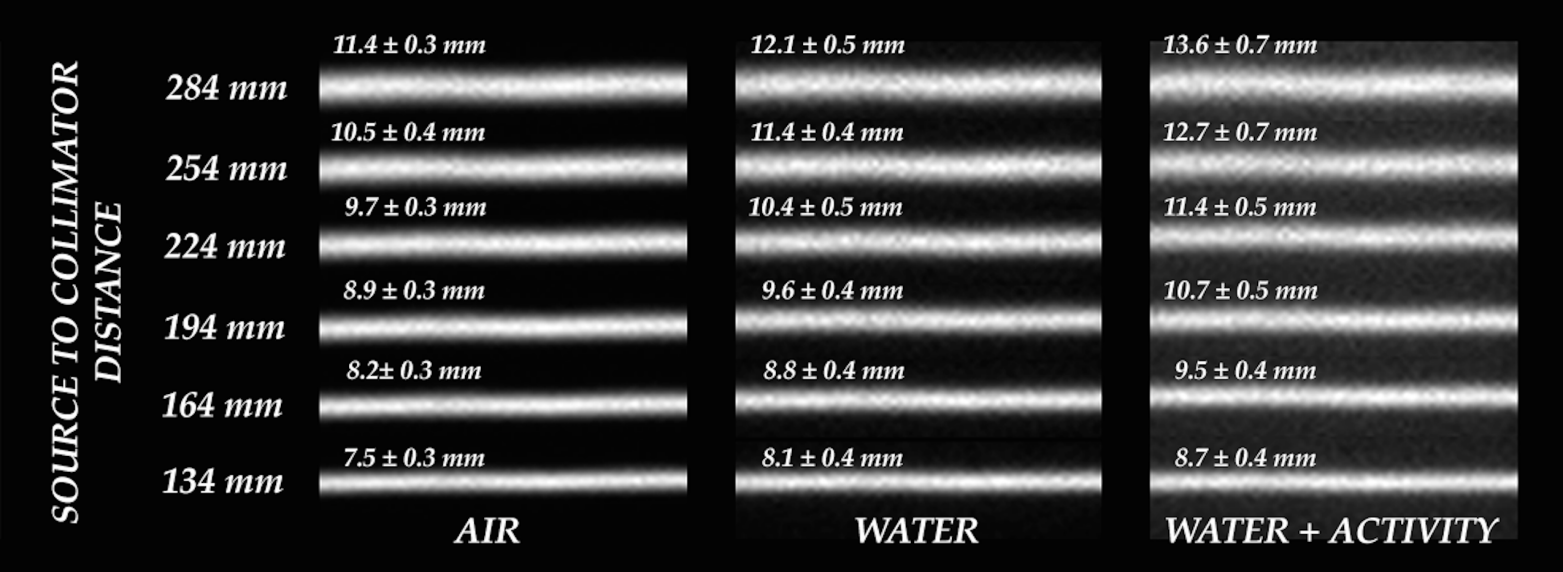
Overall comparison between zoomed-in details of line sources acquired in all conditions.

Figure 5 shows an overall comparison between zoomed-in details of line sources acquired in all conditions.

It should be noted that:

- the direct method, as expected, demonstrated higher *σ*, Cv and mean cost values than the other two methods.
- the FWHM values obtained using global and local interpolation methods were nearly identical, and *σ* and Cv were also very similar for local and global interpolation methods.
- the mean cost calculated using splines is significantly lower than when the other methods are used (at least 50% less than the cost of global interpolation).
- all previous observations were also valid in the case of different scattering conditions.
- as expected, the FWHM increases with source-to-collimator distance (radius).
- all the methods used revealed a cost that decreased in proportion to source-to-collimator distance.

## Discussion

The direct method is very quick and easy but it proves more “costly” than the other methods. The resulting FWHM does not differ significantly from the others because it is obtained as the mean of a number of profiles. It is important to use the means of multiple profiles if the direct method is the only one available, but the high Cv should discourage the use of this method.

The results obtained using local and global interpolation were nearly identical in terms of FWHM, *σ* and Cv but different in term of mean cost. Local interpolation is therefore more reliable than global interpolation when a small sample of profiles is chosen.

It should be noted that the widely used gaussian interpolation is much more time-consuming (about 25 times) than spline calculation. Given its lower cost and higher computational speed, the splines method is a very good choice for calculating FWHM from a static image. Where images are used qualitatively, reported differences in FWHM (between local and global approaches) are irrelevant. If images are also used for a quantitative approach, it is mandatory to have a FWHM value as reliable as possible. Therefore, in this latter situation, the spline method seems to be a better choice.

When the FWHM obtained using analytical calculations is compared with the results of the splines method, the difference, in air, range from about 3% to 5% (up to 0.5 mm for the largest radius); the greater the difference, the larger the radius. As expected, this trend becomes worse with a greater degree of scattering (even considering, as in our data, a low background activity concentration). For example the difference between the experimental FWHM with background and analytical resolution ranges from 13% to 15% (up to 1.7 mm for the largest radius). The software presented in this work is able to quantify this uncertainty effectively in terms of *σ*, Cv and cost.

## Conclusions

Three mathematical methods for assessing the experimental resolution obtained from static data were inputed and tested on Phantom-derived data. Local interpolation using splines proved more reliable and faster than the usually adopted gaussian interpolation (the global method).

An open source package for calculating analytical and experimental FWHM was developed in MATLAB/Octave and proved effective in assessing both FWHM and its uncertainty. A similar PHP web-based application was also developed for open access. Both tools enable a graphical and numerical comparison of experimental and analytical FWHM. These programs are freely available at: http://www.rad.unipd.it/fwhm/.

## Additional Information and Declarations

### Data Deposition

The following information was supplied regarding the deposition of related data: Matlab/Octave program: http://www.rad.unipd.it/fwhm/.
